# Supplementary material for: Copper Ionophores as Novel Antiobesity Therapeutics
Source: Molecules. 2020 Oct 27;25(21):4957. doi: 10.3390/molecules25214957 (PMC7672559; doi:10.3390/molecules25214957)
Supplement: Supplementary file 1 [file molecules-25-04957-s001.zip › Text S1.docx]

**Supplementary Figure 1:** **Disulfiram moderates weight gain in mice fed a high-fat diet (HFD) in a dose-dependent manner.** **(A)** (i) Ten-week old mice fed HFD supplemented with disulfiram (0.05% w/w and 0.125% w/w) (DSF) (*n*=3) progressively gained less weight than mice fed either HFD alone (HFD) (*n*=4) or normal chow (NC) (*n*=4). Weight change as a percentage over the 8-week feeding regime is shown. (ii) Food intake in all mice was measured weekly and expressed as (g) of feed consumed/day/mouse. **(B)** (i) Ten-week old mice fed HFD supplemented with disulfiram (0.25% w/w, 0.5% w/w and 1% w/w) (DSF) (*n*=4) all displayed acute weight loss over a 2-week period, in comparison to mice fed either HFD alone (HFD) (*n*=3) or normal chow (NC) (*n*=4). (ii) Food intake in all mice was measured weekly and expressed as (g) of feed consumed/day/mouse. Results represent mean ± SD. (**p* < 0.05; ***p* < 0.01; *****p* < 0.0001).

**Supplementary Figure 2: Histological examination of major organs in mice fed disulfiram.** Representative H&E stained sections of brain (A), kidney (B), spleen (C), lung (D) and heart (E) from mice fed either normal chow (NC) (*n*=4), HFD (HFD) (*n*=4), or HFD supplemented with disulfiram (0.05% w/w) (DSF) (*n*=4) after 8-weeks. Total magnification was either 40X(i) or 400X (ii).

**Supplementary Figure 3: Disulfiram at high concentrations in a high-fat diet (HFD) does not cause liver pathology in mice.** Representative H&E stained sections of liver from mice fed either normal chow (NC) (*n*=4), HFD (HFD) (*n*=4), or HFD supplemented with disulfiram (0.25% w/w, 0.5% w/w and 1% w/w) (DSF) (*n*=4) after 14-days. Total magnification was either 40X or 200X as shown.

**Supplementary Figure 4: Disulfiram does not modulate systemic zinc or iron in mice.** Inductively coupled plasma mass spectrometry (ICP-MS) was used to measure zinc (A) and iron (B) concentrations in the liver (i), brain (ii), lungs (iii), pancreas (iv), spleen (v) and kidneys (vi) of mice fed either normal chow (NC) (*n*=4), high-fat diet (HFD) (*n*=4), or HFD supplemented with disulfiram (0.05% w/w) (DSF) (*n*=4) for 8 weeks. Results represent mean ± SD and are shown as µg/g wet weight. (**p* < 0.05; ***p* < 0.01).
