# Supplementary figures and images for: Copper Ionophores as Novel Antiobesity Therapeutics
Source: Molecules. 2020 Oct 27;25(21):4957. doi: 10.3390/molecules25214957 (PMC7672559; doi:10.3390/molecules25214957)

A

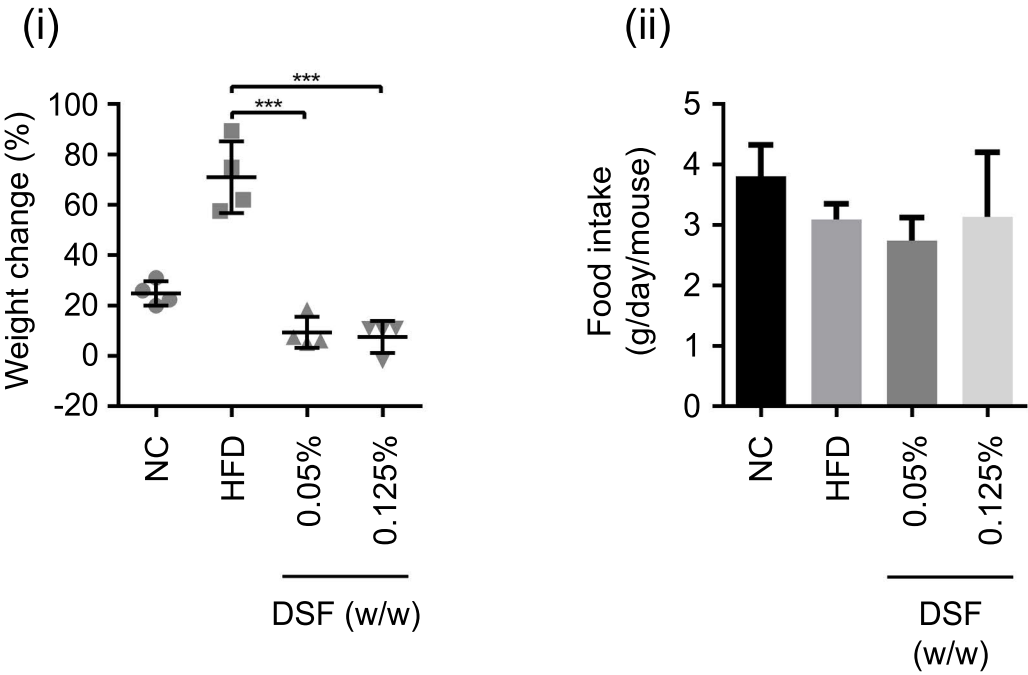

B

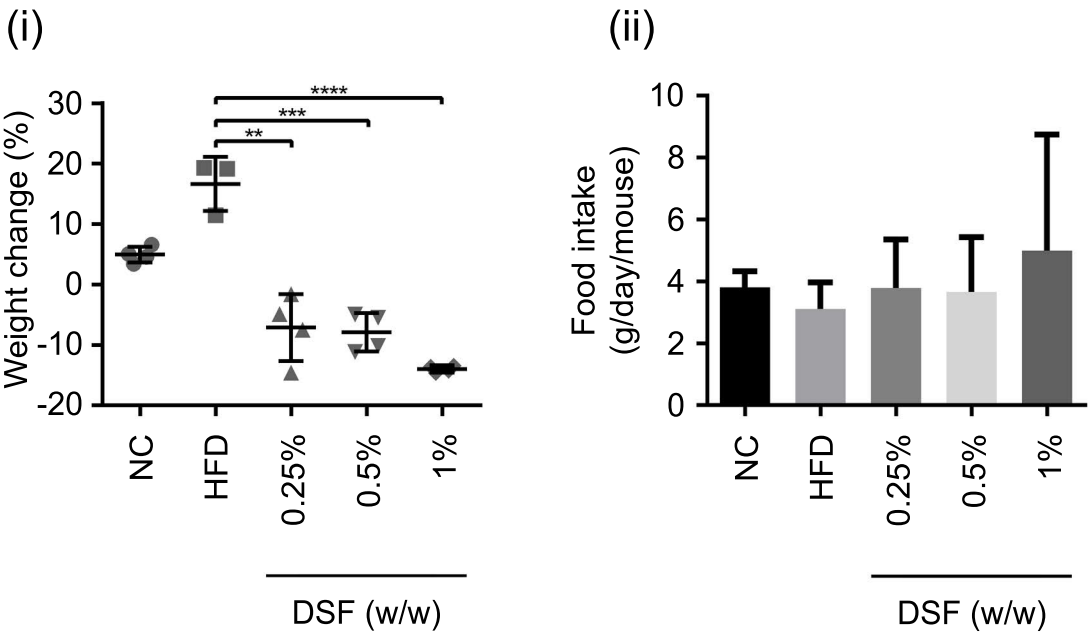

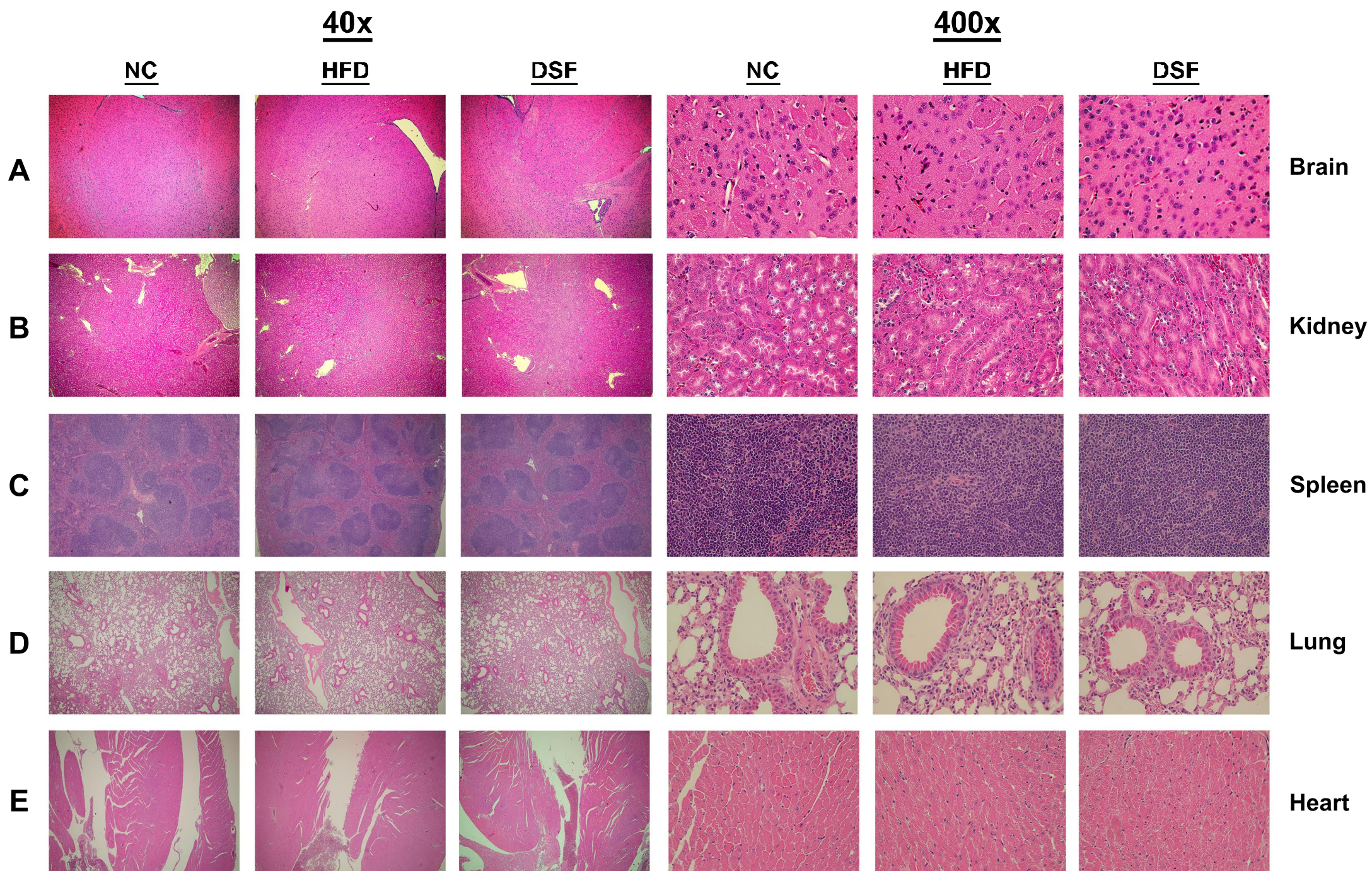

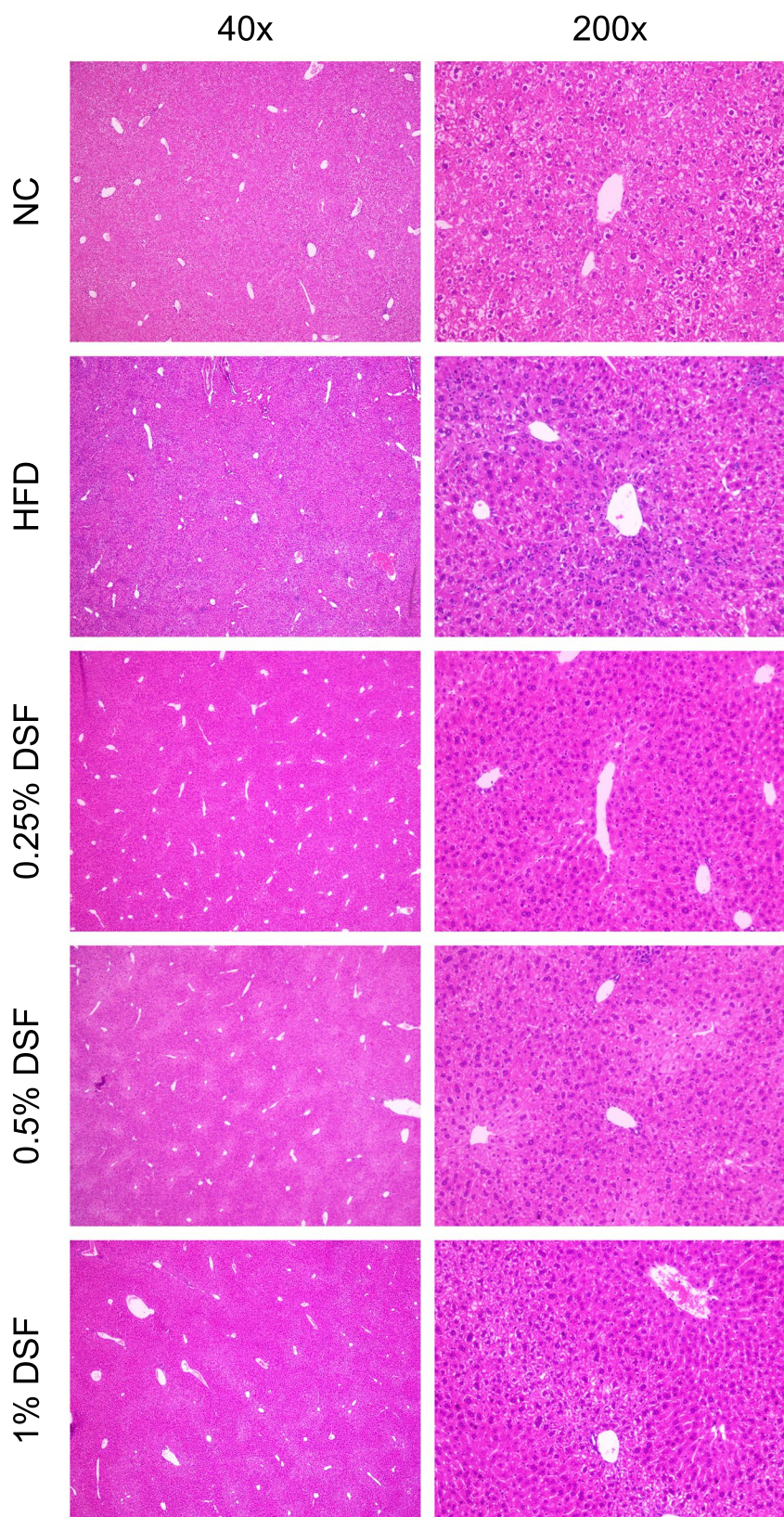

**A**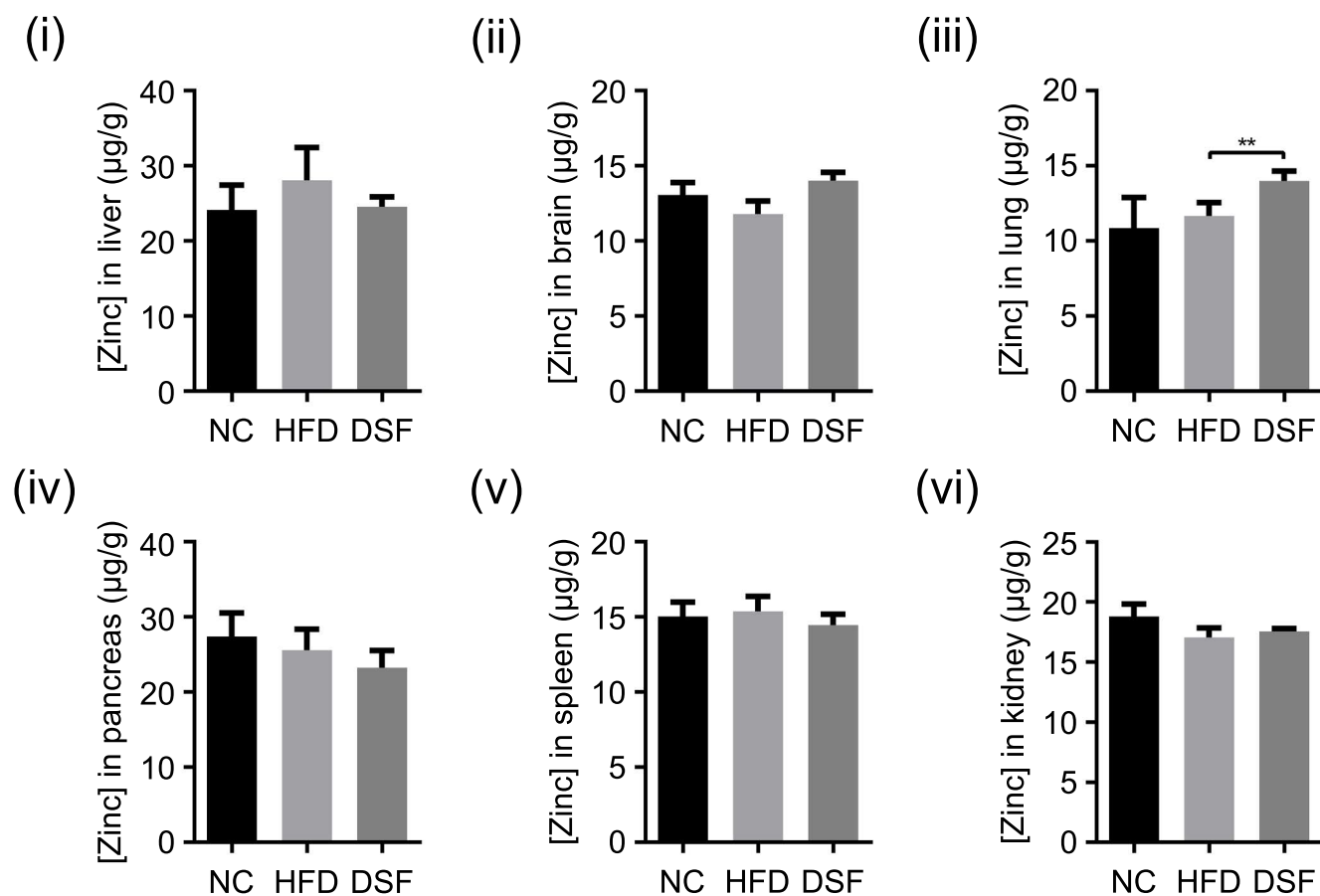**B**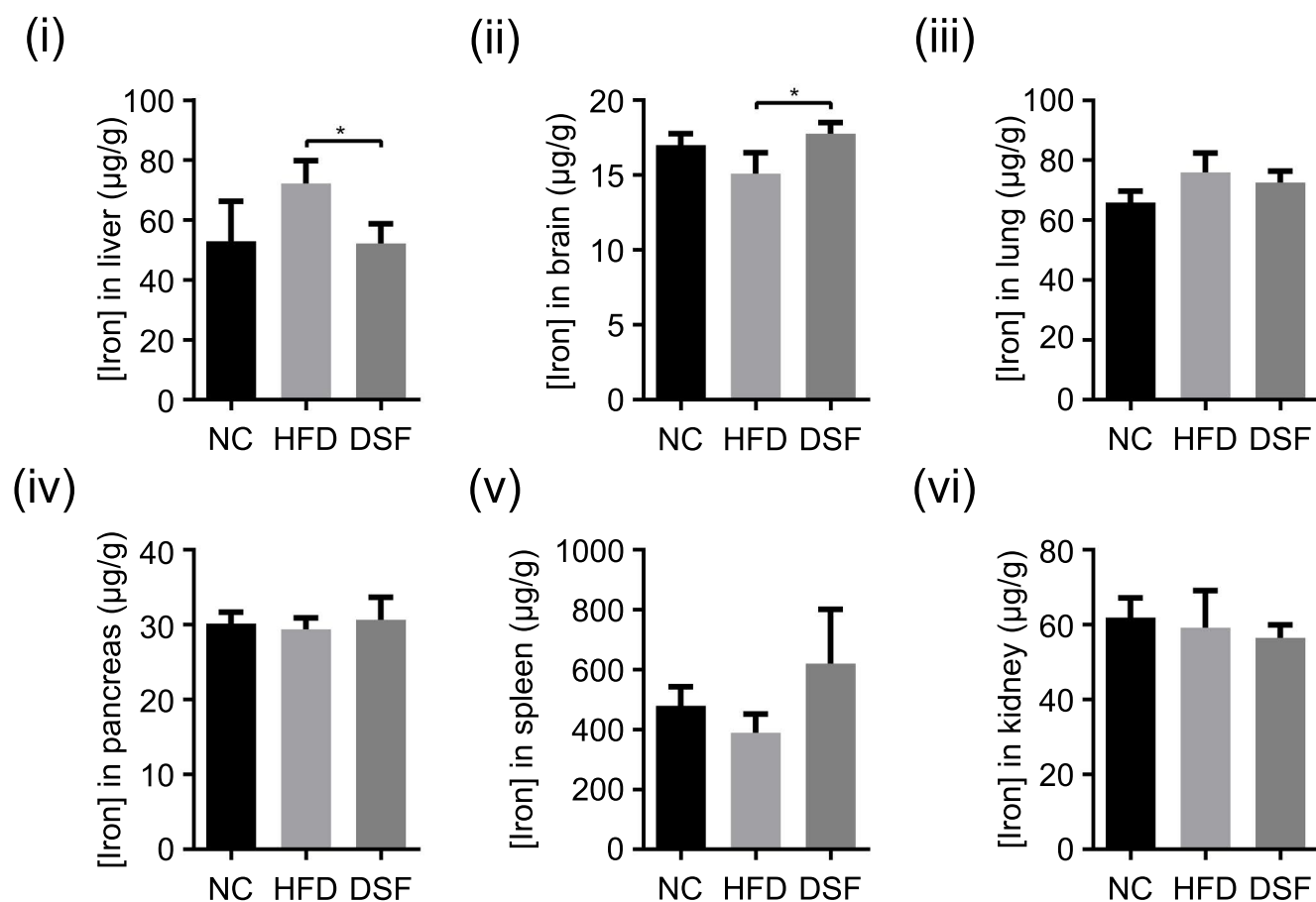

Supplement: Supplementary file 1 [file molecules-25-04957-s001.zip › Figure S1.pdf]
